# Redefining Health-Related Fitness: The Adaptive Ability to Foster Survival Possibilities

**DOI:** 10.1186/s40798-025-00826-9

**Published:** 2025-03-06

**Authors:** Natalia Balague, Consuelo San Gabriel, Robert Hristovski

**Affiliations:** 1 Complex Systems in Sport Research Group, Institut Nacional d’Educació Fisica de Catalunya (INEFC), University of Barcelona (UB), Barcelona, Spain; 2 Center of Personalized Phytotherapy, Barcelona, Spain; 3 Complex Systems in Sport Research Group, Faculty of Physical Education, Sport and Health, Ss. Cyril and Methodius University, Skopje, 1000 Macedonia

**Keywords:** Health-related fitness, Biological fitness, Psychological fitness, Social fitness, Emotional fitness, Mental fitness, Sex differences, Grand-mothering hypotheses, Entropy measures, Biological intelligence, Functional diversity potential

## Abstract

Fitness has been dominantly defined in terms of physical conditioning components. Under such definition, males tend to outperform females in strength, speed, aerobic or anaerobic capacity when compared at the same age and training status. However, females have a higher life expectancy, which in humans is related to higher biological fitness. Using the paradox of sex differences in fitness—where males have higher physical fitness but do not have a higher life expectancy—the aim of this opinion paper is to (a) highlight the multidimensionality of fitness, and (b) redefine health-related fitness, drawing on key fitness goals in biology: adaptability and survival. The redefinition of health-related fitness as the “adaptive ability to foster survival possibilities” encompasses synergies across physical, mental, psychological, emotional, social and subjective dimensions, while embracing the diversity of human characteristics, including sex, gender, age, somatotype, vital state, disability, disease and wellbeing, among others.

## Key Points

• Health-related fitness is commonly defined in terms of physical conditioning components.

• Although males may outperform females in key physical fitness dimensions and its physiological correlates they do not have a higher life expectancy than females.

• In biology, fitness is related to survival and adaptability. Accordingly, a redefinition of health-related fitness is proposed: the adaptive ability to foster survival possibilities.

• Embracing physical, mental, psychological, emotional and social fitness dimensions, the new definition is inclusive and adapts to personal characteristics such as sex, gender, age, vital state, somatotype, disability, disease and wellbeing, among others.

## Introduction

The term “fit” is an adjective that defines a state that meets the required purpose. As purposes change with contexts, fitness is defined and measured differently across fields. Health-related fitness has been defined as one’s ability to execute daily activities with optimal performance, endurance, and strength with the management of disease, fatigue, and stress and reduced sedentary behavior [1]. More recently, the American College of Sports Medicine (ACSM) has defined it as a set of attributes that people have or achieve related to their ability to perform physical activity. These attributes generally include cardiovascular endurance, muscular strength, muscular endurance, flexibility, and body composition [2]. The most common exercise prescription guidelines summarize these physical conditioning attributes to strength and aerobic capacity [3, 4].

In contrast to this fragmented and reductionist understanding of the fitness concept, in Biology fitness is defined as the ability of species to survive and reproduce in its environment [5]. Indeed, evolutionary biology has shown that the fittest is not necessarily the strongest, nor the fastest or endured, but the most adaptive and functionally diverse [6]. Since environmental challenges, personal contexts and vital states are multidimensional, and change over time, the definition of fitness cannot be restricted to physical capabilities and body composition [7]. Instead, it should integrate all possible properties that promote survival and adaptability, encompassing synergies across physical, mental, psychological, emotional, social and subjective dimensions while embracing the diversity of human characteristics.

Using the paradox of sex differences in fitness -where males outperform females in key physical fitness dimensions but do not have a higher life expectancy- the main aim of this opinion paper is to highlight the multiple dimensions of individual fitness and redefine the health-related fitness concept, drawing on insights of evolutionary biology.

### Are Women Less Fit Than Men??

The study of sex differences has gained relevance in recent years as a key step towards improving human health and pursuing scientific excellence [8]. In particular, understanding its impact on health sciences is crucial for tailoring adequate assessment systems and intervention programs for all populations.

Especially after puberty, physiological and anatomical sex-related differences increase, and according to commonly evaluated fitness indicators, males outperform females in key physical fitness dimensions such as muscle strength, power, speed, and endurance for the same age and training status [9]. However, paradoxically, it is widely recognized that females have a higher life expectancy [10, 11], which is related to a higher biological fitness. The fact that preterm infant girls have a higher chance of survival than boys [12], and that females have greater life expectancy even under very harsh conditions [13], confirms the survival advantage of women, modulated by behavioral and social factors [14].

The postreproductive longevity of females, that distinguish humans from all other primates, is explained by two different adaptive proposals: the grandmother [15] and the mother hypotheses [16]. The grandmother hypothesis states that it is selectively advantageous for females to stop reproducing in order to enhance their daughters’ fertility by helping to raise grandchildren, which allows families to grow larger and healthier. The mother hypothesis, in turn, finds advantageous for women to cease reproduction and concentrate their resources and energy in raising the children already produced. According to the grandmothering hypothesis, long postmenopausal lifespans have contributed to the survival of Homo sapiens over other hominids [15]. However, although women exhibit greater longevity compared to men, they experience greater morbidity particularly late in life [14]. They live 5 years in poorer health, corresponding to 4.7% of the total years lived compared to men [17]. Females have a higher lifetime prevalence of anxiety (M: F 1:1.7) and mood disorders (M: F 1:1.9) compared to males, whereas autism (M: F 3.3:1), attention deficit/hyperactivity disorder (ADHD) (M: F 2.2:1), and antisocial behavior are more common in males. The sex-related differences in the microstructure of subcortical grey matter regions could also be associated with mental health issues known to vary by sex, including the extent of depression, anxiety, ADHD, and antisocial behavior symptomatology [18]. The mortality-morbidity paradox, which is typical of the human species, has various speculative explanations [14].

### Multidimensionality of Sex-Related Fitness Properties

Sex-related differences contributing to the limits of human performance and fitness have been reported in the scientific literature [9, 19, 20]. Table 1 contrasts common physical fitness properties, prevailing dominantly in males (left column), with other less commonly assessed fitness-related biobehavioral properties prevailing dominantly in females (right column). The table highlights a clear sexual dimorphism and complementarity of certain fitness properties, which is considered as an advantage for the survival possibilities at the level of the species [21]. However, at the individual level, the physical components that prevail in males represent just one more fitness dimension that contributes to adaptability and survival. Actually, life expectancy is a complex phenomenon shaped by the intricate interplay among diverse individual fitness dimensions, biological predispositions and socio-behavioral factors [13].

**Table 1:** Sexual dysmorphism of physical and biobehavioral fitness-related properties. Left column: common fitness properties and their physiological correlates that prevail dominantly in males. Right column: biobehavioral fitness-related properties prevailing dominantly in females. Extant biobehavioral properties of males are not in the table with aim to enhance the contrast

Fitness-related properties that prevail in males	Fitness-related properties that prevail in females
Muscle mass [9, 19]	Interoception [42]
Low body fat [9, 19]	Emotional ability [43]
Muscle strength, power, speed, and endurance [9, 19]	Attention [48,49]
Proportion of type II fibers (fast twitch) [29]	Sensorial perception [42]
Oxidation of carbohydrates and proteins [35,36]	Immune response and adaptive immunity [54, 55, 56]
Anaerobic capacity [37]	Chromosome stability [48] and telomere length [49]
Aerobic capacity [9, 19]	Fluctuations of gonadal and non-gonadal hormones [46,47]
Blood volume, stroke volume and cardiac output [19]	Complexity in patterns of gene expression [46]
Arterio-venous oxygen difference [19, 38]	Neural network density [55]
Heart and lung size [9,40]	Interhemispheric brain communication [52,53]
Diameter airways, smaller lung volumes [40]	Social skills [44]
Alveolar-capillary membrane diffusing capacity [40]	Resistance to mitochondria damage [50]
Hemoglobin levels [39]	Efficiency to store and preserve energy lost [50]
Thermoregulation during exercise [41]	Physiological complexity [66,67]

Different fitness dimensions have been identified in the literature. Psychological fitness has been defined as the integration and optimization of cognitive processes and abilities, behaviors, and emotions to positively affect performance, well-being^1^, and response to stress [22]. Social fitness, a term used in military contexts, is defined as the ability to engage in productive personal and professional relationships, positively interact with unit and command networks, and use resources that promote overall well-being [23]. Also coming from the military context, emotional fitness refers to the ability to comprise and facilitate the emotional resilience system [24]. In relation to it, mental fitness is defined as an ability to maintain and improve cognitive function, emotional well-being, and resilience [25]. Fitness domains and their multiple dimensions are interconnected [26]. They are heavily relying on biological factors, related to human’s sexual reproduction and differentiation, and modulated by behavioral and social constraints [27].

Regarding strength, the magnitude of the female’s sex inferiority depends on age, muscle group and muscle contraction type [28]. The differences are not attributed to lower voluntary activation but to lower muscle mass and proportion of type II (fast twitch) fiber areas [29]. Woman have a greater proportion of type I (slow twitch) fibers area, superior intrinsic mitochondrial respiration [30], and exhibit greater resistance to fatigue in some types of exercise performed at the same relative intensity [31]. This enhanced resistance to fatigue has been associated to higher blood flow and muscle perfusion [32], higher density of capillaries per unit of skeletal muscles and higher beta oxidation [33, 34]. In contrast, they oxidize less carbohydrates and proteins [35, 36] and have lower anaerobic capacity [37]. With respect to the aerobic capacity, they have smaller blood volume, smaller stroke volume, cardiac output [19] and reduced arterio-venous oxygen difference [38]. Even if corrected for lean body mass, VO_2_max, the gold standard index of cardiorespiratory fitness, is inferior in women due to their lower hemoglobin concentration [39]. With respect to ventilation, they have smaller diameter airways, smaller lung volumes, lower maximum expiratory flow and lower alveolar-capillary membrane diffusing capacity [40]. In addition, their larger sex hormonal (estrogens and progesterone) fluctuations, may influence negatively substrate metabolism and thermoregulation during exercise [41].

In contrast to physical fitness, other fitness dimensions contributing deeply to survival and adaptivity have been less explored and compared between sexes (see Table 1, right). In general, women are more perceptive of the external and internal environment [42, 43], although social and emotional cues often lead them to mistrust bodily internal signals, making it more likely to attribute changes to emotions in oneself and others [44]. They have more accurate and developed sensorial perceptions [45], better verbal description of colors, sounds, odors, tastes or touch compared to males [43]. Their reproductive function is physiologically more complex due to variations in gene expression in both reproductive and non-reproductive organs [46], along with fluctuations in the levels of gonadal and non-gonadal hormones over time [47]. Importantly, while X chromosomes are generally stable during lifetime, Y chromosomes are gradually lost with successive somatic cell mitosis during male lifetimes. Higher ratios of X0 cells vs. XY cells seem related to shorter individual lifespans [48]. In addition, telomere length, which is associated with biological age, is longer in females than in males [49].

To insure the fetal development and carry out a successful birthing, nursing and caring, women have more efficient mechanisms to preserve energy and muscle mass [50]. Relying on oxidation of lipids, they preserve glucose for neuronal and placental function and spare proteins necessary for organ function. Carbohydrate reliance in men are also related to evolutionary adaptation (e.g. defense, hunting), as glucose is the fuel for anaerobic high-exertion muscle activity. The larger lipid storage and higher mitochondrial activity of female may reduce the transmission of metabolic disorders. In particular, their pattern of fat distribution (subcutaneous fat), accumulated around the hips, thighs, and buttocks is somewhat protective from cardiovascular and metabolic diseases, specially before menopause. In contrast, the androgenic pattern of fat storage (visceral fat) around the abdomen is more harmful because it is metabolically active and releases pro-inflammatory substances, which are associated with a higher risk of cardiovascular and metabolic diseases [51].

The success of females in sexual reproduction entails fostering complex social networks among family members, and cooperation with males, other females and grandmothers. Developing muscular strength and non-specific conditioning would be unnecessary physiological investments for females who often possess enhanced perception, social skills, and sophisticated behaviour towards youngsters and members of the group [44]. Indeed, the study of the human brain networks suggest that females present more dense neural networks and better interhemispheric communication than males [52, 53], which is related to advantages in social motivation, attention and memory tasks. Males in turn, show greater interhemispheric communication, related to motor and spatial abilities.

During gestation, the immune system of females complexifies notably because it has to tolerate genetically different individuals, provide them with nutrients and oxygen, discard their waste, get ready for parturition, prepare for lactation, and nurture newborns [54]. The acquired antigens may persist years beyond pregnancy; behaving as a dynamic, versatile pool, and can be replaced by new ones in subsequent pregnancies. This strong innate and adaptive immunity protect woman from malignant cancer and antibody responses [55, 56]. During pregnancy, and mediated by the microglia activity, new network synapses are created to support nesting, nurturing, nursing, and promptly responding to stimuli from the young [57]. These mothering behaviors have a deep impact in offspring survival and reproductive success [58, 59]. Since they persist long after weaning, producing permanent changes in females [60, 61], they may contribute to their higher life expectancy.

### Redefining Health-Related Fitness

Considering the multiple human properties that can contribute to survival and adaptivity, physical conditioning components (e.g., strength, speed, or endurance) maybe viewed as just a few expressions of individual health-related fitness. In other words, similar to the proposition that human intelligence transcends purely logical and mathematical abilities, it is posited that human fitness cannot be solely encapsulated by physical conditioning metrics. As a multifaceted array of psychological, physiological, behavioral, social and subjective dimensions, we advocate for a redefinition of health-related fitness as follows: *adaptive ability to foster survival possibilities*^2^. The new definition, focused on adaptive and evolving abilities is inclusive and embraces differences, including but not limited to, sex, gender, age, vital state, somatotype, disability, disease and wellbeing. Even a disabled person or a person with a disease can be considered fit, not only because of the subjective dimension of fitness—where a disabled person or someone with a disease may experience a good fitness state, while a healthy person may experience poor fitness without any objective evidence—but also because they successfully adapt to hard life challenges and survive with the best possible quality of life within the particular circumstances.

In the domain of biological intelligence, conceptualized as a subset of cooperative-competitive intelligence, Hristovski and Balagué [62] have introduced the notion of functional diversity potential to elucidate the survival capacities of an individual, both within the context of sport and in broader life circumstances. The authors conceptualize biological intelligence as the capacity to avoid or extricate oneself from states characterized by diminished functional diversity. The biological intelligence operates through flexible compensatory synergies across the dimensions that constitute functional diversity potential. For instance, diminished endurance may be offset by enhanced coordination or anticipatory skills [62]. Also, any unforeseen disruption, such as the occurrence of an epidemy, has the potential to decrease functional diversity, however, the acquired immune response, confers protection against subsequent infections. This suggests that the human organism retains adaptive information, enabling it to endure a wide array of extreme environmental conditions and effectively navigate novel challenges.

The functional diversity potential may be reduced by competition with other organisms (e.g. opponents, bacteria, virus) or other environmental constraints (e.g. climate conditions). The ability to create reciprocally compensatory synergies of effective and efficient functional solutions to the challenges of the environment cannot simply rely on strength and endurance [7]. The ability of the organism to adapt to changing environments creating functional and flexible synergies depends on its diversity potential and its metastability [63], a crucial property to remain responsive and adaptive. In relation to the concept of health-related fitness examined in this paper, compensatory processes may manifest through the engagement in psychologically or socially meaningful and fulfilling activities, notwithstanding the presence of physical disability or multimorbidity.

Under the new definition, fitness does not correspond to specific biobehavioral capabilities and concrete quantitative values. Thus, as proposed by the theory of biological intelligence, it can be assessed through entropy measures [64] that quantify the synergetic compensation across various activity dimensions. These measures can operate across multiple domains, including sociology, psychology, and biology, encompassing scales from the cellular to the societal. Further research is needed to develop entropy measures for assessing the health-related fitness state and its evolution over time.

The non-decreasing diversity potential principle constitutes the fundamental goal of biological intelligence, defined by an organism’s capacity for rapid adaptation to changes and challenges, thereby enabling species to persist within their natural environments [65]. In this sense, the concepts of biological intelligence and health-related fitness are straightly related.

Consequently, health-related fitness facilitates prompt recovery from states of reduced functional diversity by fostering the development of novel solutions within dimensions unaffected by disability. As a result, the new definition of health-related fitness gains a significantly broader conceptualization and manifestation, extending beyond fragmented physical, psychological, or social abilities.

## Data Availability

Not applicable.

## Footnotes

1 Within the framework of the theory of cooperative and competitive intelligence [62], one can be in a state of wellbeing even with multiple comorbidities, as long as they are able to successfully compensate for them with healthy activities, such as socialization, adapted physical activity or spiritual practices, among others.

2 Reproduction, a key concept in biological fitness that reflects how well an individual is adapted to its environment in terms of genetic contribution to future generations, has not been included in the definition because this work focuses on fitness at the individual level rather than the species level.

## Abbreviations

ACSM: American College of Sports Medicine
CCI: Cooperative-competitive intelligence
